# A fraction of diet-derived microRNAs present in plant extracellular vesicles remain detectable after gastrointestinal digestion and shows epithelial uptake in a CaCo-2 cell model

**DOI:** 10.1016/j.fochms.2026.100450

**Published:** 2026-09-18

**Authors:** Miguel Cifuentes-Acebal, Adria Vilalta, Ximena Terra, Yvan Devaux, Torsten Bohn

**Affiliations:** a Nutrition and Health Research Group, Department of Precision Health, Luxembourg Institute of Health, Strassen, Luxembourg; b Doctoral School in Science and Engineering, University of Luxembourg, Esch-sur-Alzette, Luxembourg; c Universitat Rovira I Virgili, Dept. Bioquímica I Biotecnologia, MobioFood Research Group, Tarragona, Spain; d Cardiovascular Research Unit, Department of Precision Health, Luxembourg Institute of Health, Strassen, Luxembourg

**Keywords:** Bioavailability, Exosomes, Food-bioactives, Gut, XenomiR, PELN, Bioaccesibility

## Abstract

Diet constitutes a source of exogenous miRNAs that are detectable in humans. Source, stability to digestion, and cellular uptake of diet-derived miRNAs are debated topics. Here, we studied the stability of cauliflower miRNAs to cooking (100 °C, 20 min) and gastrointestinal digestion (static INFOGEST 2.0), their possible cellular uptake/secretion by CaCo-2 cells, and the role of plant extracellular vesicles (PEVs). miRNAs were assessed by qPCR. The role of PEVs was evaluated via dynamic light scattering, electron microscopy, and immunoblotting. Most RNA was degraded after digestion (99.1%). However, a fraction of miRNAs in PEVs (bol-miR172a, bol-miR156a, bol-miR164b, bol-miR1030i) remained detectable (0.05–0.24%). PEVs were also detected after digestion, which caused aggregation and decreased particle numbers. Finally, nano-encapsulation, concentration and exposure time appeared associated with miRNA uptake (40-fold compared with control). Data for secretion was inconclusive. Overall, we showed that a small fraction of miRNAs associated with PEVs remained detectable following digestion.

## Highlights

• A fraction of cauliflower miRNA (<1%) was detected after gastrointestinal digestion.
• Some extracellular vesicles (1–10%) were stable to gastrointestinal digestion.
• miRNA contained in extracellular vesicles showed more resistance to digestion.
• Time, concentration, and encapsulation favor miRNA uptake by the gut epithelia.
• Secretion of miRNAs seems promoted when contained within extracellular vesicles.

## 1 Introduction

Diet is widely recognized as one of the main factors regulating health and disease through chronic exposure (Cifuentes et al., 2024; Marx et al., 2021). Many bioactive components of diet have been subject to research based on these grounds (Bohn et al., 2021). While secondary plant metabolites have attracted considerable interest, other dietary constituents have been neglected. One of these are nucleic acids, which have only recently gained some attention by the scientific community (Del Pozo-Acebo et al., 2021). Specifically, microRNAs (miRNAs) have gained interest as novel components of diets, whose full impact on human health is still unknown (Jia et al., 2021).

miRNAs are small non-coding RNA molecules, ranging from 21 to 24 nucleotides in length and which are involved in transcriptional and post-transcriptional regulation (Del Pozo-Acebo et al., 2021; Jia et al., 2021). These molecules are ubiquitous to multicellular organisms, present at high concentrations in various food matrices, and are more resistant to degradation than larger RNA molecules (i.e. mRNA) (Borges & Martienssen, 2015; Jung et al., 2010; Tarver et al., 2012). The reasons for this increased resistance are multiple and range from association with RNA binding proteins to secondary structure properties (Winter & Diederichs, 2011; Zhang et al., 2012). Considering their widespread and relative resistance to degradation, it should not be surprising that studies have detected exogenous miRNAs within the human body (Zhang et al., 2012). The “dietary xenomiR hypothesis” proposes that diet constitutes a source of exogenous bioactive miRNA that are detectable in the human body (Witwer, 2012). Thus, diet-derived miRNAs could have a potential use as biomarkers of food intake and further have direct implications on human health. However, research on this topic has been scarce (Del Pozo-Acebo et al., 2021).

Progress thus far has mostly focused on identifying potential sources of diet-derived miRNAs (e.g., rice, cabbage, or milk) (Del Pozo-Acebo et al., 2021; Diez-Sainz et al., 2024). Unfortunately, key aspects including stability to kitchen procedures such as cooking, or gastrointestinal digestion (GID), key for their ensuing potential bioactivity, are far from being understood. Even though some research on digestion and stability of diet-derived miRNAs has been conducted, this has usually been limited due to weak experimental designs (i.e. use of stool samples to evaluate stability, omission of key steps in GID, etc.) (Diez-Sainz et al., 2024; Jia et al., 2021; Liang et al., 2012; Philip et al., 2015; Qin et al., 2022). A similar knowledge gap exists regarding their cellular uptake (Tambaro et al., 2025). When data is available, it usually has been produced employing miRNAs devoid of potential protective elements such as extracellular vesicles, which may also impact cellular uptake (Del Pozo-Acebo et al., 2021). In addition, limitations in the domain of cellular uptake are of particular concern, as multiple studies have failed to detect systemic circulation of diet-derived miRNAs in human plasma or other biofluids (Dickinson et al., 2013; Witwer et al., 2013; Witwer & Hirschi, 2014). Moreover, multiple studies have indicated that initial reports of circulating xenomiRs might have originated from bioinformatic pipelines limitations, background noise from qPCR, or cross-contamination during miRNA sequencing, as recently reviewed (Tambaro et al., 2025). However, lack of detection in systemic circulation does not exclude stability to digestion and even biologically relevant functions such as local effects within the gut epithelia (Tambaro et al., 2025). Thus, the xenomiR hypothesis and potential stability of diet-derived miRNAs remain elusive topics.

In this regard, extracellular vesicles (EVs), which are small (approx. 30–150 nm in diameter) vesicles containing a lipid bilayer have emerged as a promising vehicle for diet-derived miRNAs (Lopez de Las Hazas et al., 2023). EVs are loaded with various molecules, such as proteins and nucleic acids that can transmit intercellular signals across tissues (Hessvik & Llorente, 2018). In plants, similar structures to animal EVs exist. These are typically referred to as plant EVs (PEVs) (Martinez Fajardo et al., 2025). PEVs have been proposed as the main vehicles of diet-derived miRNAs (Del Pozo-Acebo et al., 2021; Suharta et al., 2021). According to some authors, the encapsulation of miRNAs in PEVs could protect them from the action of physicochemical and enzymatic processes during GID (Del Pozo-Acebo et al., 2021; Tambaro et al., 2025). However, little research has focused on the effect that nano-encapsulation has on diet-derived miRNA stability and cellular uptake (Del Pozo-Acebo et al., 2021; Tambaro et al., 2025). The latter is thought to happen through endocytosis, as it would normally occur for endogenous miRNAs in other tissues (Del Pozo-Acebo et al., 2021). Nonetheless, it is unknown whether PEVs play a role here, or alternatively, miRNA absorption is mediated through other mechanisms such as membrane diffusion or paracellular transport (Del Pozo-Acebo et al., 2021). These combined knowledge gaps significantly hinder progress in the study of miRNAs as food bioactives (Jia et al., 2021; Tambaro et al., 2025).

Therefore, we conducted a series of experiments that aimed to shed light on the dietary xenomiR hypothesis. The focus rested on endogenous cauliflower (*Brassica oleracea* var. *botrytis)* miRNAs, as recent work has shown how miRNAs from this species are resistant to GID (Chapado et al., 2021). Particularly we employed bol-miR172a as a reference miRNA, as it has been described to survive GID in mice (Liang et al., 2014). In addition, a panel of other GID stable miRNAs (bol-miR164b, bol-miR156a) (Cavallini et al., 2018; Diez-Sainz et al., 2024; Huang et al., 2018; Zhang et al., 2012) and highly expressed cauliflower miRNAs (bol-miR1030i) (Chowdhury et al., 2021) was developed. We used a recently developed protocol to purify diet-derived miRNAs from gastrointestinal digesta (Cifuentes Acebal et al., 2025), and to study the effects that GID has on miRNA stability, as well as the role of PEVs. We also assessed the cellular uptake of diet-derived miRNAs and their basolateral sequestration using the Caco-2 cell line, a model of the human gut epithelia. We thus strived to provide a better understanding on diet-derived miRNAs, particularly on the conditions that affect their stability, and the mechanisms responsible for their cellular uptake and transport, both subjects that have been identified as critical for moving forward research on diet-derived miRNAs, including their potential health aspects (Tambaro et al., 2025).

## 2 Methods

### 2.1 Chemicals reagents and consumables

Unless otherwise stated, all chemicals were of molecular biology grade or superior. Chemicals were purchased from Sigma Aldrich (St. Louis, MO, US), including KCl, KH_2_PO_4_, NaHCO_3_, NaCl, MgCl_2_(H_2_O)_6_, (NH_4_)_2_CO_3_, CaCl_2_(H_2_O)_2_, porcine pepsin from gastric mucosa (powder, 250 U/mg, Cat. P7000), porcine pancreatin (activity equivalent to 4× USP specifications, Cat B1750), bovine bile (B3883), as well as chloroform, isopropanol 99.5%, and ethanol 100%.

mirVana mimics (cel-miR39-3p and bol-miR172a), Lipofectamine™ 3000, Qubit™ RNA High Sensitivity Assay kit, Qubit™ microRNA Assay kit, Pierce BCA protein assay kit, non-DEPC treated water, Dulbecco's Modified Eagle Medium (DMEM) supplemented with GlutaMAX™, *RNAsecure ™*, *Opti* MEM™ media, Gibco premium fetal bovine serum (FBS), non-essential amino acid solution (100×) from Lonza™ (Basel, Switzerland), 0.8 μm sterile nylon filters (Sartorious, Germany), sterile 12-well PET non-coated plates from Nunc (Roskilde, Denmark), sterile transwell inserts with 0.4 μm PET membrane and 0.9 cm^2^ surface and their accompanying plates from Falcon, NuPAGE LDS sample Buffer 4×, Novex Tris-Glycine SDS Running Buffer 10×, NuPAGE Transfer Buffer 20×, Pierce TBST 20× Buffer, Pierce SuperSignal West Femto, PVDF membranes, were purchased from Thermo Fisher Scientific (Waltham, MA, US). Dulbecco's Phosphate Buffer Saline (DPBS) 1× w/o Ca & Mg, and w/o phenol red, and penicillin/streptomycin (100×) with l-glutamine from Capricorn Scientific were purchased to Westburg Life Sciences (Leusden, Netherlands).

### 2.2 Acquisition of samples

Cauliflower (*Brassica oleracea* var. *botrytis*) was bought in a fresh (4 °C) pre-cut state package at a local supermarket (Delhaize, Strassen, Luxembourg). The tissue was ground to small particle size upon arrival at the laboratory. Samples for which RNA extraction was to be carried out without further treatment (raw matrix) or that were only to be thermally incubated (simulated cooking) were divided into 50 mg aliquots. Alternatively, aliquots of 4 g were prepared for those samples to be subject to GID. Samples used for extracellular vesicle extraction were ground and aliquoted at 4 g. All samples were frozen and stored at −80 °C until further use.

### 2.3 Sample processing

Samples underwent diverse physicochemical processing steps to mimic cooking and GID (Supplementary Table 1). Vegetable cooking was conducted by adding treated nuclease free water to the samples and incubating in a heated block (100 °C, 20 min). Later, the samples were cooled down to 4 °C and the excess water was removed. Nuclease free water for cooking was used aiming to increase reproducibility and to limit any degradation other than that of thermal stress alone, leaving any enzymatic or chemical degradation for gastrointestinal digestion. GID of samples was done according to the static INFOGEST 2.0 digestion model. Fig. 1 shows the different processing steps and the number of biological replicates.

**Fig. 1:**
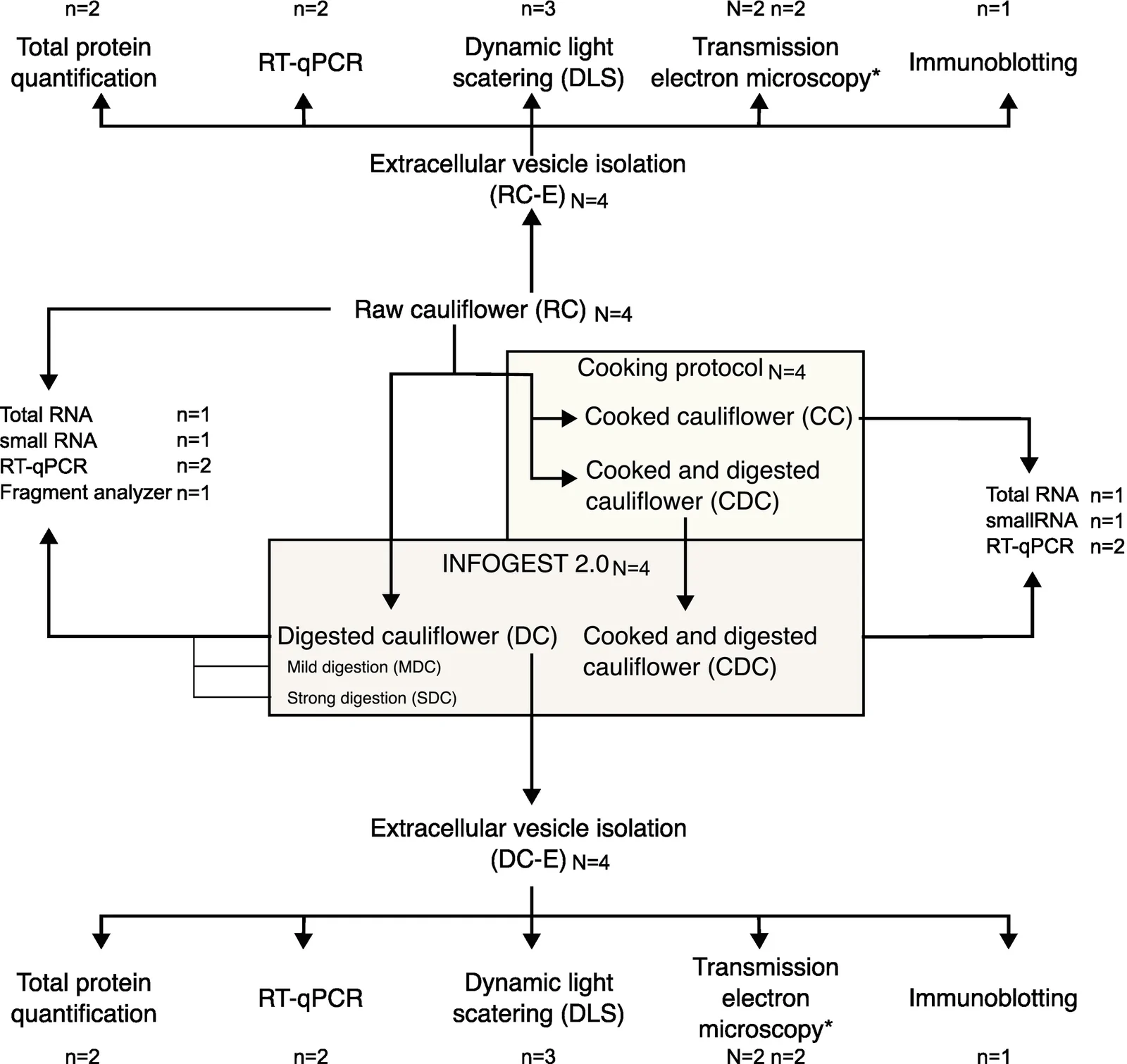
Summary workflow on the production of the cauliflower derived samples and parameters that were assessed for each of them. The capital letter N indicates the number of biological replicates produced at each instance while the small capital letter n is shown next to an experiment to indicate the number of technical replicates. The asterisk (*) indicates that only two biological replicates instead of four were measured for a given test.

Different conditions were carried out based on the intersection of the treatments described above (Supplementary Table 1). First, samples that were not subjected to any modification are hereinafter referred to as raw cauliflower (RC). Other samples were subjected to thermal incubation but no GID, referred to as cooked cauliflower (CC) (Fig. 1). Alternatively, some samples were subjected to GID but not to thermal incubation, termed digested cauliflower samples (DC). Finally, the combination of thermal incubation (done first, 100 °C, 20 min) and GID (INFOGEST, following cooking) was termed cooked and digested cauliflower (CDC). For subsequent analysis (i.e., extracellular vesicle analysis), only RC and DC were retained (Fig. 1).

### 2.4 Sample digestion

GID was done in accordance with the INFOGEST 2.0 protocol, a consensus model of static digestion (Brodkorb et al., 2019), with minor modifications (Cifuentes Acebal et al., 2025) (Supplementary Table 2). In brief, samples were first incubated with a simulated gastric fluid for 2 h. Then, a second incubation with simulated intestinal fluid was carried out. Both incubations were done at 37 °C and a horizontal shaking speed of 100 rpm. A final volume of 26 ml was reached per 4 g of initial sample. The final digesta was centrifuged to remove any large non-bioaccessible particles and the supernatant (∼10 ml) was filtered through a 0.8 μm filter to produce the bioaccessible phase. Said phase was frozen at −80 °C until further use. For extracellular vesicle purification from GID, the sample was immediately utilized, avoiding freeze/thaw cycles that could compromise the vesicles' integrity. For additional details on the in vitro digestion of samples, we refer the reader to the original article describing the method in full (Brodkorb et al., 2019).

In addition to the standard model described above, modifications were introduced to recreate both milder (MDC) and stronger (SDC) digestive conditions (Supplementary table 2) (Fig. 1). In the case of the mild digestive conditions, an increase in pH in the gastric phase to 4 was performed together with a reduction to half of the concentrations of pepsin in the gastric fluid, as well as bile salts and pancreatin in the intestinal phase. Alternatively, stronger digestive conditions were simulated via an enzyme concentration of 1.5 x compared to the standard digestion. In addition, the pH of the gastric phase was decreased to 2 and the shaking bath speed increased from 100 rpm to 140 rpm for the entire digestion for an approximate doubling of kinetic energy. Other GID parameters remained unaltered from the standard protocol.

### 2.5 Development of a miRNA panel

The miRNAs employed throughout the manuscript were selected according to two criteria. First and foremost, primer compatibility and availability with the RT-qPCR system employed for detection (Qiagen miRCURY, see Section 2.13). Second, we referred to a publication in which cauliflower miRNAs were recently sequenced (Chowdhury et al., 2021) and investigated whether some of these had also been reported to be stable to GID in other species. Consequently, we identified two miRNAs, bol-miR164b and bol-miR156a (Cavallini et al., 2018; Diez-Sainz et al., 2024; Huang et al., 2018; Zhang et al., 2012). In the sequencing experiment by Chowdhury and colleagues, an exceptionally high expression of bol-miR1030i was reported and thus we also retained this molecule, anticipating that GID might fully degrade miRNAs with low expression. Finally, we included a fourth molecule (bol-miR172a) based on our previous findings related to stability in addition to reports from others (Cifuentes Acebal et al., 2025; Liang et al., 2014).

### 2.6 Cell culture

CaCo-2 (ATCC HTB-37) cells were used as a model for epithelia in the small intestine. The cells were cultured for four passages (26–29). Cells were split in a 1:6 dilution when 80% confluent. Seeding of cells into plates or transwell inserts was done at a concentration of 1*10^5^ cells/cm^2^. A pore size of 0.4 μm was used in the transwell. Cells were allowed to grow in a confluent monolayer for 21 days. Culture media consisted of DMEM containing GlutaMAX and supplemented with non-essential amino acids (5%), antibiotics (penicillin and streptomycin, 5%) and FBS (10%). Cells were incubated at 37 °C, and 5% CO_2_ content and the media was changed every 3–4 days.

### 2.7 Cellular uptake and secretion

For the uptake analysis, the media in the wells was changed at the start of the experiment. Incubation conditions and their preparation are shown in supplementary tables 3 and 4. In short, miRNAs, artificially nano encapsulated miRNAs, bulk RNA extracts, and PEVs were presented to the cells at different concentrations and timepoints to study whether selected miRNAs could be detected in association with the cells or even secreted after a short (4–6 h) incubation period basolaterally. If a transwell was used, the test media was added to the apical site, and the basolateral compartment was washed twice with PBS and replenished with DMEM at the beginning of the incubation. Lipofectamine 3000 was used as a transfection reagent following manufacturer's guidelines. Data on the conditions of incubation with lipofectamine is given in the supplementary tables 3 and 4. The TEER (transepithelial electrical resistance) potential was measured using a Millicel ® ERS-2 Voltohmmeter (Merck, Darmstadt, Germany) to verify the confluence of the monolayer, the plate set-up and TEER values of the different wells at the start of the incubation are displayed in supplementary table 5. A reference value of 300 Ω*cm-2 was selected as a threshold to indicate tight junction integrity and monolayer confluence (Kus et al., 2023). The concentrations for incubation were previously calculated to be equivalent. Thus, 0.16 nM, 300 ng of RNA extract/ mL of DMEM, and 0.45 nM in the transwell insert all correspond to approximately 1*10^11^ copies of bol-miR172a. The concentration of miRNAs in PEVs could not be matched due to low yield and technical constraints. The estimated number of miRNAs loaded per condition (and the equation employed for the calculation) can be found in supplementary tables 3 and 4.

Following incubation, we collected the media and performed three consecutive washes of the cells with PBS. In the following, QIAzol (Qiagen) was applied to each well to lyse the cells and stabilize the RNA. Apical and basolateral media, together with cellular RNA content were then stored at −80 °C for later use. Briefly, the experiments for uptake/association (Supplementary table 3) included the incubation of non-polarized CaCo-2 cells with a miRNA mimic (bol-miR172a) at different concentrations (0.016, 0.16, and 1.6 nM), as well as the use of RNA extracts (10, 25, and 75 μl of RC at 300 ng/μl), and PEVs from RC-E and DC-E (25 μl). In addition, Lipofectamine 3000 was added to some miRNA mimics and bulk RNA to simulate nanoencapsulation of PEVs. For studies of basolateral secretion, polarized CaCo-2 cells were used. In this case, bol-miR172a was incubated with the cells at a concentration of 0.045 and 0.45 nM. RC-E was incubated at different volumes (75 and 175 μl) and for DC-E 200 μl were used. In addition, an extra timepoint (6 h) was included. Supplementary Table 4 contains further information on the conditions tested, the concentration of miRNA and the preparation of the conditions.

### 2.8 Plant extracellular vesicle purification

PEVs were purified using the ExoEasy Maxi Kit (Qiagen, Hilden, Germany). When starting from a solid matrix (RC-E), an input amount of 4 g of pre-homogenized plant matrix was mixed with two volumes of PBS. Next, a 20-min incubation period with shaking at room temperature was conducted to promote PEV release to the PBS. After incubation, the samples were filtered with a syringe using glass wool to remove large particles. Once the liquid phase containing PEVs (∼8 ml) was obtained, the samples were passed through a 0.8 μm nylon filter and then the protocol was continued following manufacturer's guidelines. For liquid matrices (DC-E), the protocol started here, and 10 ml of input volume was used. In short, PEVs were mixed with 1 volume of binding buffer and transferred to a membrane affinity spin column. Later, wash buffers were applied to the column, and the final elution of extracellular vesicles was performed in 400 μl of elution buffer XE.

### 2.9 Zeta-potential, size determination, and protein content

The PEV extracts were analyzed by dynamic light scattering (DLS) using a Zetasizer Ultra Red Label (Malvern Panalytical, Malvern, UK) to study particle size, particle concentration, and zeta-potential. For this purpose, the PEV extracts were diluted 1:100 in PBS in a final volume of 1 ml for the analysis.

Study of the protein content in PEVs was carried out using a Pierce™ BCA protein assay, following the manufacturer's guidelines. In short, 25 μl of undiluted PEVs sample were mixed with 200 μl of BCA working solution and incubated for 30 min at 37 °C. Afterwards, absorbance at 562 nm was measured at 25 °C in a SpectraMax M5^e^ (Molecular devices, San Jose, US). Extracts were then immediately used for immunoblotting, and a small aliquot was frozen at −80 °C for electron microscopy imaging.

### 2.10 Extracellular vesicle visualization

Visualization of PEVs was achieved through transmission electron microscopy (TEM). A standard negative staining protocol using 2% uranyl acetate was employed for labelling the PEVs as described elsewhere (Rikkert et al., 2019). The micrographs were obtained by the Scientific & Technical Resources Service of the Universitat Rovira I Virgili (Tarragona, Spain).

### 2.11 Immunoblotting

Purified PEVs were used directly for protein analysis (i.e., no lysis of vesicles). A 30 μl of PEV's solution volume (in Buffer XE) was selected for DC-E samples, while RC-E samples were adjusted to 10 μg of total protein. Denaturing of proteins was conducted for 5 min at 75 °C. Then, the protein solution was loaded into a 4–20% gradient gel together with X4 Laemmli buffer. The electrophoresis was run for 2 h at 100 V.

The immunoblotting step was conducted using common PEV surface markers (TET8 and PEN1/SYSP121) (Supplementary Table 6). The transfer was conducted using a PVDF membrane in a tank system (Mini Protean, Bio-Rad) for 60 min at 100 V. Skim milk (5%) in Tris-buffered saline and Tween 20 surfactant (TBST) was used as blocking buffer. No-stain agent was used to label total proteins in the membrane. Blocking was done by incubating the membrane for 1 h in blocking buffer. The primary antibody labelling was achieved following overnight incubation at 4 °C. The secondary antibody was incubated for 1 h at room temperature. Staining of the membrane was done using a chemiluminescent substrate (SuperSignal West Femto and Super Signal West Atto), and the chemiluminescent signal (∼425 nm) was captured using an iBright Imaging System (ThermoFisher Scientific).

### 2.12 RNA purification

Total RNA purification, including miRNAs, was done following different techniques according to the starting material (i.e. PEVs, cells, cell culture supernatant, plant tissue, or GID). For the GID bioaccessible phase, we used the miRNeasy Serum/Plasma Advanced Kit (Qiagen) with modifications for GID extraction as described previously (Cifuentes Acebal et al., 2025), using an input volume of 600 μl. The same kit but following manufacturer recommendations and 600 μl input volume was used to extract RNA from cell culture supernatant. PEV RNA was extracted using ExoRNEasy Midi and Maxi kits (Qiagen) for RC-E and DC-E, respectively, following manufacturer guidelines. The remainder of the samples (i.e., plant tissue, cells) were extracted using the miRNeasy mini-Kit (Qiagen), following manufacturer recommendations for each sample type. Details on the procedure for each sample type are given below.

For extraction from the plant tissues, 50 mg of fresh tissue were homogenized using disposable plastic pestles after adding QIAzol. When working with cells, these were homogenized by vortexing the tubes containing QIAzol for 1 min. Cell culture media and GID bioaccessible phase (600 μl) were disrupted using a lysis buffer. Regarding RC-E and DC-E, the column containing the PEV isolate was incubated with 700 μl QIAzol to extract RNA instead of eluting the nano vesicles. RNA extraction was conducted following manufacturer guidelines. After disruption, samples were spiked-in with cel-miR39-3p (3.5 μl at 33.2 nM) and incubated at room temperature for 5 min. Final elution was done in RNase-free water.

RNA extracts were evaluated using a Nanodrop 1000 spectrophotometer (ThermoFisher Scientific). A Qubit fluorometer 4 (ThermoFisher Scientific) was used to measure concentrations of total RNA (High Sensitivity assay) and small RNAs (microRNA assay). The nucleotide size distribution of specific samples (i.e., RC and DC) was studied using a fragment analyzer (Agilent, Santa Clara, CA, US) with an HS RNA kit (Agilent) to investigate the size distribution of RNA molecules. Unless otherwise reported, concentrations are reported as ng of RNA per mg of fresh cauliflower.

### 2.13 Reverse transcription and qPCR

Reverse transcription was done using the miRCURY LNA RT kit (Qiagen) and a C1000 Touch Thermal Cycler machine (Bio-Rad, Hercules, CA, US). All samples were spiked with 0.5 μl of a synthetic miRNA molecule (UniSp6) prior to RT as indicated in the miRCURY LNA kit protocol. This molecule was used as a control for the reverse transcription. Two approaches were followed for the reverse transcription. For samples with cell-associated RNA (i.e., a matrix containing cells) and exosomal samples, 200 ng of total RNA were loaded for each reaction. When cell-free RNA was extracted (i.e., digesta), we used an input volume of 4.5 μl of RNA for the reaction. The input volume for each sample type had been previously optimized (Cifuentes Acebal et al., 2025). Master mix and cycling conditions were prepared according to manufacturer guidelines.

Quantitative PCR was done in a Bio-Rad CFX96 Touch Real-Time PCR Detection System, using the miRCURY LNA miRNA PCR assays (Qiagen). Samples were diluted 60 times (30 in the case of PEVs) prior to the PCR. The qPCR reaction was set up according to manufacturer guidelines. In short, 3 μl of cDNA were added to 7 μl of master mix, and the mixture was incubated for 40 cycles. The list of primers used in the reaction is given in Supplementary table 7. The RT-qPCR was conducted in accordance with the MIQE guidelines (Bustin et al., 2009), a summary of relevant data for interpretation of the qPCR results can be found in supplementary table 7. Most importantly, all primers were validated to function properly within the range provided in the current manuscript (supplementary table 7). Samples detected after 38 PCR cycles were regarded as not detected, while caution is advised in the interpretation of data for samples detected between cycles 35 and 38 (supplementary table 7).

### 2.14 Statistical analysis and software

Statistical analyses of the data were conducted with R (version 4.3.2) and RStudio (2024.04.2 + 764). The alpha-value for statistical testing was set at 0.05 (2-sided). Unless otherwise reported, data are shown as mean values ± standard deviation. Normality of the distributions and equality of variances were studied using Q-Q plots, KS tests and boxplots, respectively. ANOVA tests were used for analyzing the differences between groups when values followed parametric test assumptions (Fig. 1, Fig. 3, Fig. 4). Wen results were significant further group-wise comparisons were carried out applying the Tukey HSD post-hoc test. When a simple comparison between two groups was performed (i.e. Figs. 1E and 2) a student's unpaired *t*-test was performed. Multiple testing was adjusted using the Bonferroni method, i.e. the *p*-value was divided by the number of comparisons. If samples did not meet parametric assumptions the Kruskal-Walli's test was used. Plotting was done using the ggplot2 package from R and Inkscape was used for the final assembly of the figures. Unless otherwise reported, all percentages discussing stability of a miRNA refer to the concentration found in RC. A brief summary of the number of biological and technical replicates employed at each step, and the statistical method used for each comparison is shown in supplementary table 8. Inverted CT values employed for quantification of miRNA stability throughout the manuscript were the result of subtracting to the quantification threshold for each sample to the maximum number of PCR cycles run (40). This operation leads to higher values being associated with higher concentrations.

**Fig. 2:**
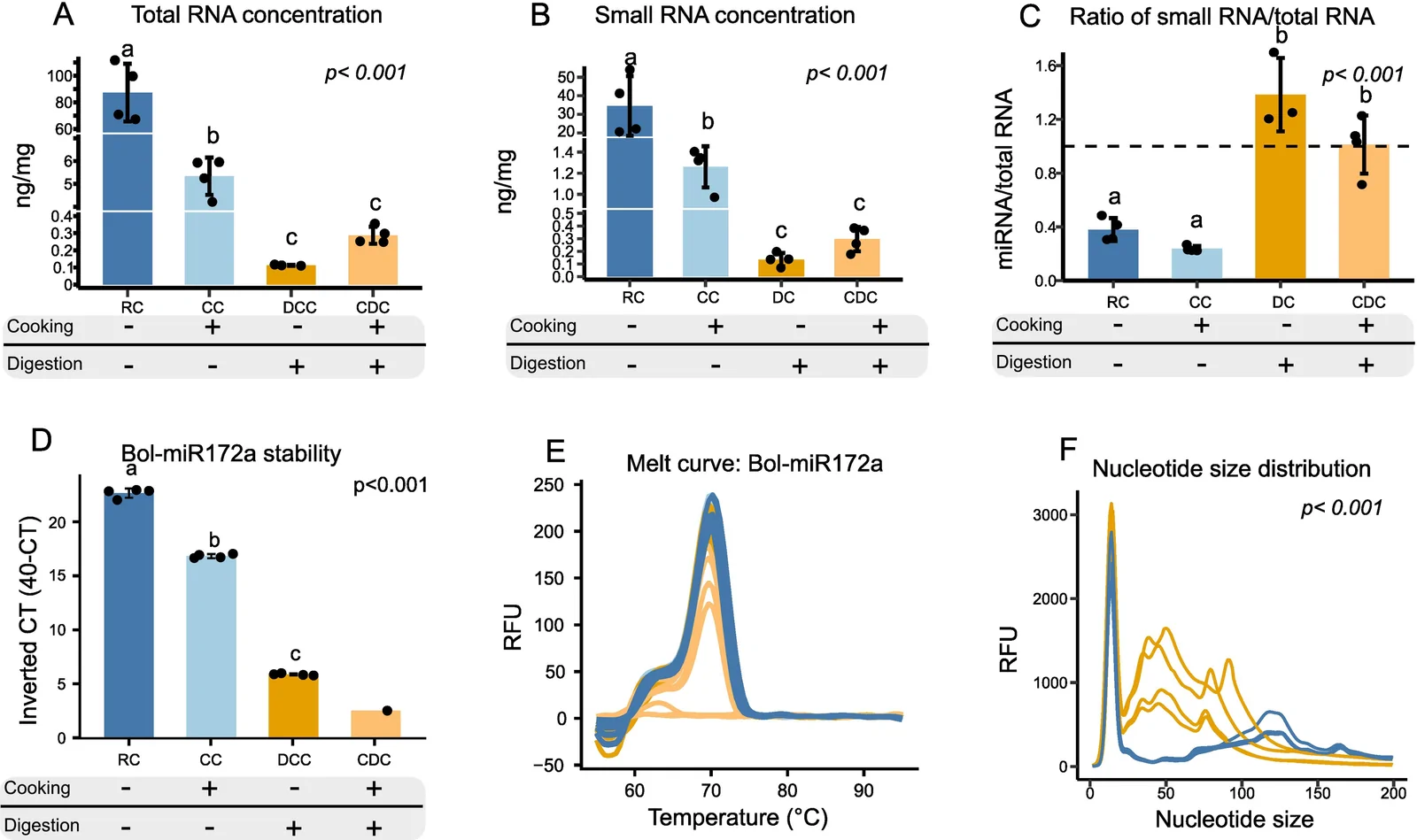
Study of the stability of cauliflower RNA, small RNA, and miRNA to gastrointestinal digestion across four treatments: raw cauliflower (RC) (dark blue), cooked cauliflower (CC) (light blue), digested cauliflower (DC) (dark orange), and cooked and digested cauliflower (CDC) (light orange). A: Total RNA contents in the different samples normalized to the amount (per mg) of cauliflower. $\eta^{2}=0.99$. *n* = 1. The y axis shows two breaking points at 0.4–4 ng/mg and 7–80 ng/mg B: small RNA concentration in the different samples normalized to the amount of cauliflower. $\eta^{2}=0.97$. *n* = 1. The y axis shows two breaking points at 0.5–0.9 ng/mg and 1.5–20 ng/mg. Note that the comparison between DCC and CC yielded a *p*-value = 0.02, above the *p* > 0.001 indicated in the panel. C: Ratio of small RNAs to total RNA in the different samples. $\eta^{2}=0.990.$*n* = 1 D: RT-qPCR data showing the expression of bol-miR172a in the different samples. Data points indicate inverted CT values (40-CT value). $\eta^{2}=0.99$ Higher values represent a higher expression. *n* = 2 E: Melt Curve data for bol-miR172a. *n* = 2 F: Fragment size distribution of nucleic acids in raw and digested cauliflower. *n* = 1. Unless otherwise stated, *N* = 4 technical replicates are indicated for each panel. Error bars represent ± SD of the mean. Letters in the panels indicate statistical significance when different. Each panel indicates the *p*-value for the comparisons (tested with ANOVA + Tukey's HSD, panel F was analyzed with a Student's unpaired *t*-test). *p*-value for each comparison shown in the top right of each panel. When multiple comparisons were performed the *p*-value was corrected using Bonferroni's method. This is the displayed value. (For interpretation of the references to colour in this figure legend, the reader is referred to the web version of this article.)

## 3 Results

### 3.1 MicroRNA detection after heat incubation and gastrointestinal digestion

Total RNA was purified from cauliflower after different stages of processing (raw, cooked, digested, and digested and cooked). The absorption spectra for the extracted RNA displayed two distinct patterns (Supplementary Fig. 1). Samples derived from the raw cauliflower matrix showed a prominent peak of absorbance at 260 nm, and a second lower peak at 230 nm. The remainder of the samples had no noticeable peak at 260 nm, which may indicate RNA loss. Absorbance at 230 nm showed high variability across samples.

Further analysis of the different conditions to understand their total and small RNA concentration was conducted. Total RNA concentration (Fig. 2A) showed that unprocessed samples (Raw Cauliflower, RC) had the highest concentration of total RNA (87.30 ± 21.74 ng/mg), with significant declines in concentration (93.90–99.98%, *p* < 0.001) after cooking (Cooked Cauliflower, CC) (5.33 ± 0.81 ng/mg), digestion (Digested Cauliflower, DC) (0.11 ± 0.00 ng/mg), or a combination of both (Cooked and digested Cauliflower, CDC) (0.28 ± 0.04 ng/mg). The same observation was applicable for the small RNA fraction (<50 nucleotides long) (Fig. 2B). A high initial concentration in RC (34.53 ± 16.01 ng/mg) was followed by a sharp decline (*p* < 0.001) in CC (1.26 ± 0.19 ng/mg), DC (0.13 ± 0.05 ng/mg), and CDC (0.29 ± 0.09 ng/mg).

When the relative abundance of small RNA content was studied compared to that of total RNA in the sample, two patterns emerged (*p* < 0.001). The first group, which was composed of RC (37.9 ± 0.9%) and CC (23.7 ± 0.02%) contained a small RNA fraction that accounted for less than 50% of the total RNA. Contrarily, when samples underwent digestion, DC (138.4 ± 27.3%) and CDC (101.3 ± 21.6%), their small RNA content increased to become the only type of detected RNA (Fig. 2C). The values over 100% were attributed to differences in sensitivity for the methods used to measure small RNAs and total RNA, leading to a technical artifact. In short, the concentration of total RNA is just below the detection limit of the Qubit High Sensitivity kit (0.2 ng/μl), which might cause a slight overestimation. Contrarily, the miRNA concentration was well within the operating range of the miRNA detection kit (0.025–150 ng/μl).

We then studied miRNA integrity during food processing, using qPCR to assess the concentrations of four endogenous cauliflower miRNAs: bol-miR172a (Fig. 2D, Table 1), bol-miR164b, bol-miR1030i (Supplementary Fig. 1, Table 1), and bol-miR156a (not detected/shown). Only bol-miR172a could be detected following a simulated GID (0.003% survival, compared to RC) (Table 2), the melt curve (Fig. 2E) shows a good quality amplification of this miRNA, with a single prominent peak at 70 °C, ruling out artifacts.

**Table 1:** Summary data on the three cauliflower miRNAs that could be measured before and after GID. Four biological replicates were prepared per condition. Data is displayed as the mean Cq value accompanied by standard deviation and 95% confidence intervals (CI). NA indicates that the miRNA could not be measured or that not enough replicates to compute the statistics were detected. *N* = 4.*n* = 2.

miRNA ID	RC			CC			DC			CDC		
	Cq	SD	CI (95%)	Cq	SD	CI (95%)	Cq	SD	CI (95%)	Cq	SD	CI (95%)
bol-miR172a	17.33	0.42	16.66–18.01	23.17	0.19	22.87–23.48	34.13	0.09	33.99–34.28	37.48	NA	NA
bol-miR1030i	29.14	0.58	28.22–30.07	30.45	0.10	30.30–30.62	NA			NA		
bol-miR164b	21.57	0.23	21.20–21.94	26.64	0.31	26.15–27.14	NA			NA		

Further evaluation of the RNA integrity was performed using capillary electrophoresis to compare RC and DC samples and evaluate their average nucleotide size (Fig. 2F). Two distinct (*p* < 0.001) profiles were revealed. In the region below 200 nucleotides, several peaks, corresponding to fragment sizes between 20 and 100 nucleotides, were detected in the digested samples. This profile was not detected in RC, where only one peak (approx.120 nucleotides) was detected below 200 nucleotides.

### 3.2 The effect of different digestion models on miRNA stability

The effect of simulated GID upon cauliflower miRNAs in the food matrix was further analyzed using different digestive conditions, with modified pH, enzymatic and bile salt concentrations as well as simulated peristalsis strength compared to the standard conditions. Between the two additional models investigated (i.e., mild, and strong digestive conditions), a statistical difference was observed in the total RNA concentration and small RNA concentration as measured by a Qubit fluorometer (Fig. 3A-B), though SDC showed a somewhat higher detection of both. Alternatively, fragment size distribution showed a similar distribution between these two models, which also showed a similar pattern to the one observed in DC samples before, i.e. a depletion of long RNA molecules in favor of molecules <200 nucleotides (Figs. 2F and 3C). In accordance with the data obtained through Qubit, a higher detection of bol-miR172a was observed in SDC samples. While no signal was retrieved for MDC samples (Fig. 3D).

**Fig. 3:**
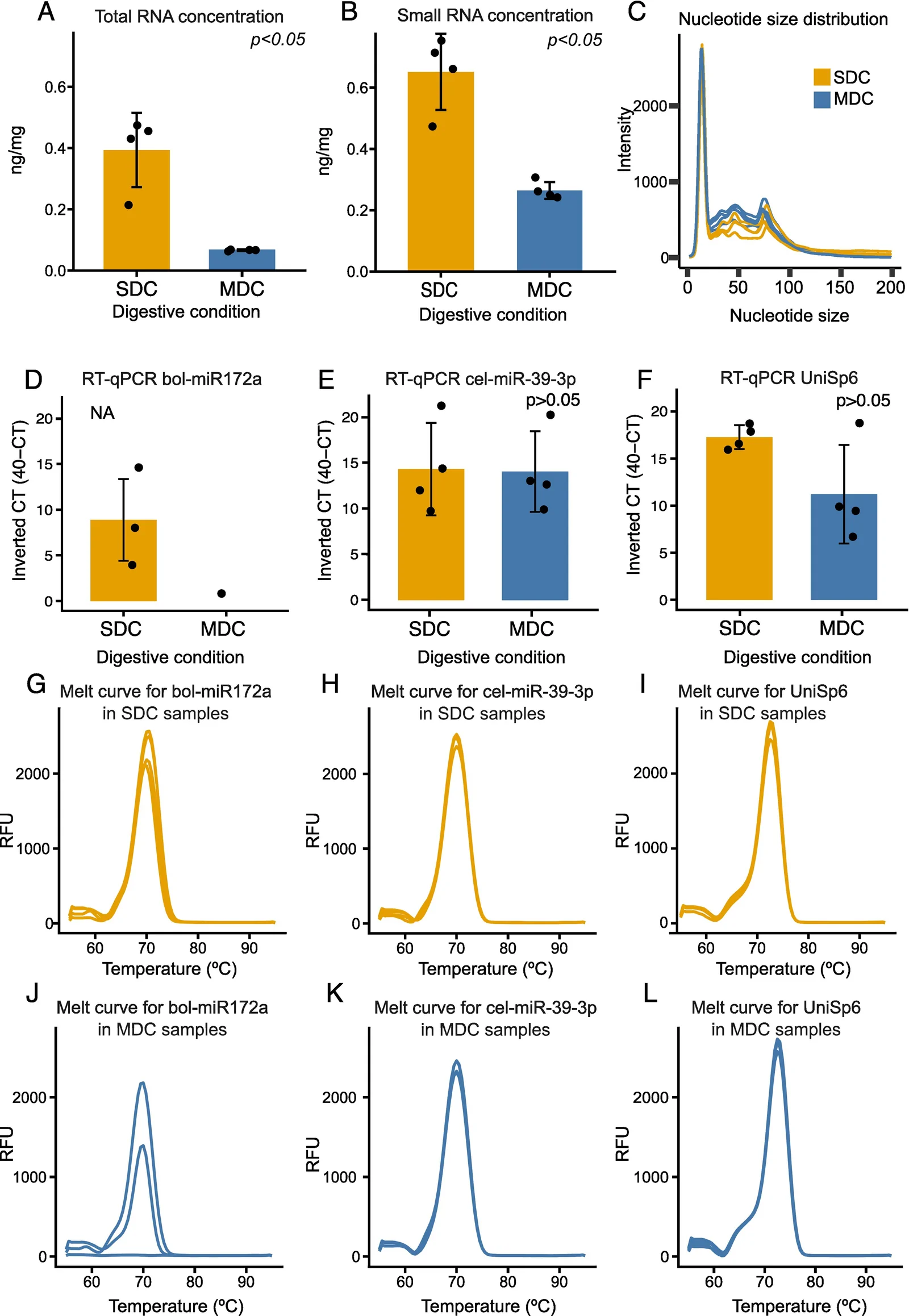
Study of the RNA contents isolated from the bioaccessible phase of a simulated gastrointestinal digestion containing cauliflower. Two digestive models based on modifications to the INFOGEST static digestion were employed. These modifications included a strong digestion (SDC, orange) and a milder digestion (MDC, blue). A: Total RNA contents of the extract as measured by a qubit fluorometer, concentration was normalized to ng of RNA per mg of cauliflower. *n* = 1 B: Concentration of small RNA molecules in the digesta normalized to ng of RNA per mg or fresh cauliflower as measured by a qubit fluorometer. *n* = 1C: Fragment size distribution (<200 nt) of SDC and MDC samples. *n* = 1 D-F: Inverted CT values (40-CT) for bol-miR172a, cel-miR-39-3p (Spike-in, RNA extraction control, expected at cycle 18–19.5) and UniSp6 (Spike-in, RT reaction control, expected at cycle 22) in SDC and MDC. *n* = 2. G-I: Melt curves for bol-miR172a, cel-miR-39-3p, and UniSp6 primers in SDC samples. *n* = 2. *J*-L: Melt curves for bol-miR172a, cel-miR-39-3p, and UniSp6 primers in MDC samples. *n* = 2. At the top left, a Student's unpaired *t*-test comparison resulted *p*-values. An NA indicates that there were not enough replicates to conduct a *t*-test. Error bars represent ±SD of the mean of *N* = 4 replicates. (For interpretation of the references to colour in this figure legend, the reader is referred to the web version of this article.)

The qPCR analysis of the samples revealed quality limitations that affected either the reverse transcription reaction efficiency, and/or the RNA extraction, as indicated by the poor recovery of our spike-in controls, which were outside of the expected range for all SDC and MDC samples (Fig. 3E-F, Supplementary Table 7). However, the melt curves indicated peaks at 70 °C for both bol-miR172a and cel-miR39-3p and of 72.5 °C for UniSp6 (Fig. 3), which fell within the expected values, discarding any issues during the qPCR step. Finally, the analysis of the absorbance spectrum of both sample types revealed a high concentration of contaminants at 230 nm that had not been observed before (Supplementary Fig. 2). Thus, these observations remain rather exploratory.

### 3.3 Characterization of PEVs before and after GID

Based on the data gathered we hypothesized that some mechanism enabled the stability of certain diet-derived miRNAs in the bioaccessible phase of GID. We theorized that the encapsulation of miRNAs in PEVs might be one of the underlying reasons accounting for their resistance to physico-chemical degradation. Therefore, we studied PEVs and their cargo's stability to GID. To do so, we characterized the size, shape, and contents (i.e., miRNA cargo, total protein, and surface markers) of these vesicles in both raw cauliflower (RC-E), as well as the bioaccessible phase of GID (DC-E).

Morphological characterization of PEVs included a classification by particle size, shape, and surface proteins. RC-E had a mean hydrodynamic diameter of 130.8 ± 63.5 nm. Meanwhile, DC-E showed a significantly higher (*p* < 0.001) mean hydrodynamic diameter of 203.3 ± 102 (Fig. 4A-B). In both cases, the polydispersity index (PDI) was close to 0.2, indicating a rather monodisperse sample (supplementary table 9). Total protein content of the extracellular vesicle extracts showed a significant decrease when RC-E (461.62 ± 26.6 μg/ml) and DC-E (40.15 ± 8.4 μg/ml) samples were compared, measured either through BCA or total protein staining on a membrane (Supplementary fig. 3A-B). Following normalization of the protein amount, using the number of particles counted with DLS (a proxy for PEV purity in the solution) did not suggest a significant difference in protein concentration, although a trend (*p* = 0.13) could be observed (Fig. 4C). The absolute number of particles which was estimated through multi-angle DLS (MADLS) decreased from 2.15*10^10^ ± 5.03*10^10^ particles in RC-E to 4.15*10^8^ ± 3.73*10^8^ particles in DC-E (supplementary table 9). Further analysis of the protein fraction through immunoblotting was aimed at two PEVs surface markers, PEN1/SYSP121 and TET8. Only SYP121 could successfully be detected in RC-E (Supplementary fig. 3C). However, it could not be detected in DC-E (Supplementary fig. 3C). The charge of the particles remained negative before and after GID (supplementary table 9).

**Fig. 4:**
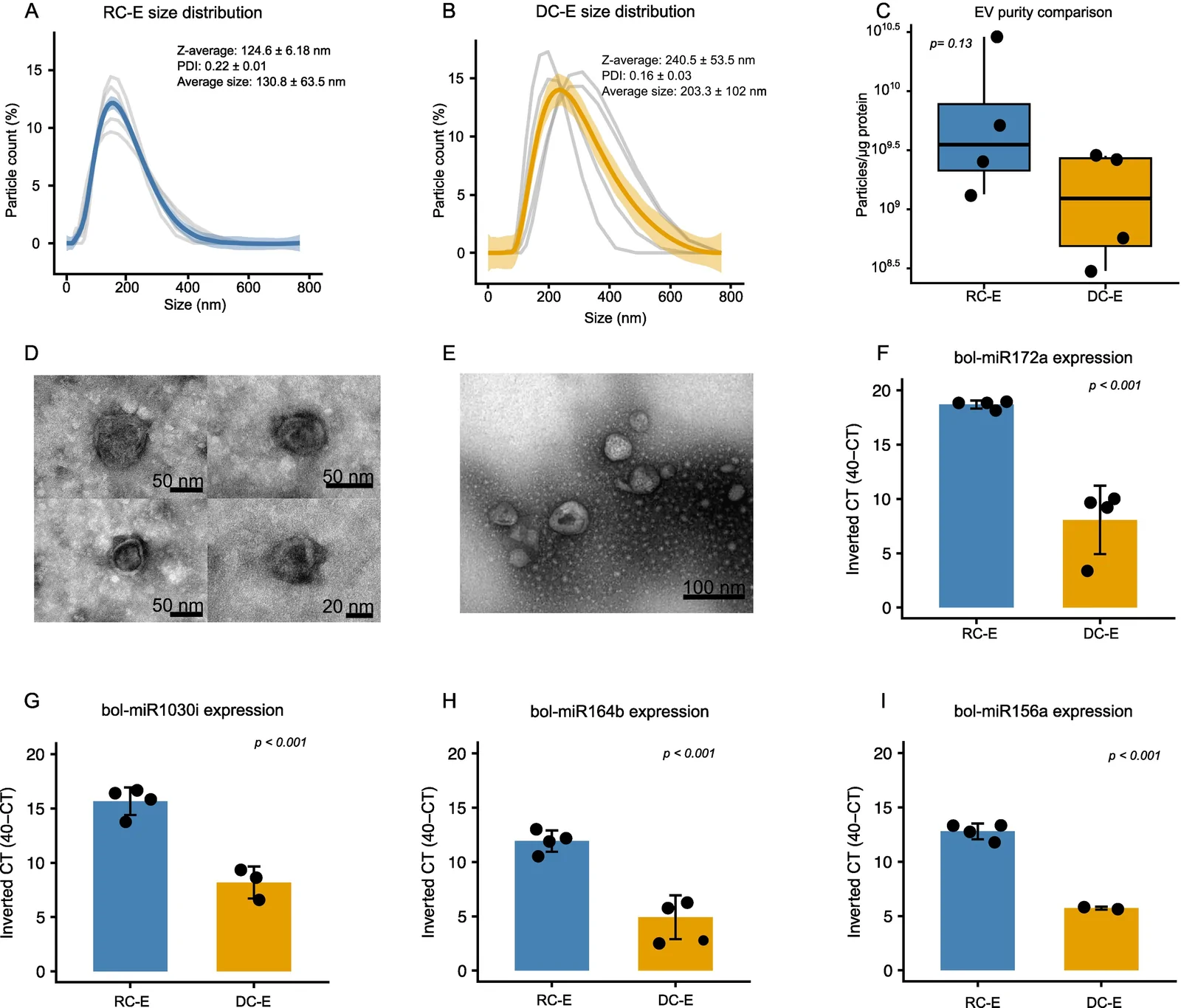
Characterization of cauliflower extracellular vesicles (EV) before (RC-E) (blue) and after (DC-E) (orange) gastrointestinal digestion. A: Particle size distribution (intensity-based measurement) as measured by dynamic light scattering (DLS) in RC-E. Data on the polydispersity index (PDI), z-average and average size are shown in the plot. In blue, the mean measurement; in grey, each individual measurement. *n* = 3 B: DLS measurement of DC-E (intensity-based), with PDI, *Z*-average and mean size displayed in the plot. In orange the mean measurement, in grey each individual measurement. *n* = 3C: Ratio comparing the number of particles in EVs isolate per μg of protein concentration, higher values indicate lower purity. D: Transmission electron micrograph (TEM) obtained from the isolated undigested EVs. The bar indicates the scale (400 x and 800 x magnification). *N* = 2, *n* = 2 E: TEM obtained from the isolate of DC- E (200 x magnification), the bar shows the scale. *N* = 2, *n* = 2. F-I: RT-qPCR data showing the expression of cauliflower miRNAs both in RC-E and DC-Eme, data is displayed as inverted CT values (40-CT). Higher values represent a higher expression. *n* = 2. The *p*-value for each RC-E and DC-E comparison is displayed on the top left corner of a panel when applicable. A *t*-test was conducted to study the differences in detection levels for each miRNA as well as for the study of the particles to protein ratio in panel C. Multiple testing was corrected by using Bonferroni's method. When error bars are shown these represent ± SD of the mean. Unless otherwise indicated, *N* = 4. Technical replicates are specified for each panel. (For interpretation of the references to colour in this figure legend, the reader is referred to the web version of this article.)

The structure and shape of particles in the extracts was analyzed by electron microscopy, which revealed that vesicles possibly containing a lipid bilayer were present in RC-E and DC-E (Fig. 4D-E). These vesicles had a spheroid shape and an approximate size of 40–100 nm in diameter. Furthermore, DC-E were usually observed in aggregates of varying size together with small lipid droplets. The miRNA cargo of RC-E and DC-E was also analyzed (Fig. 4G-I). The previously queried miRNAs originating from cauliflower (bol-miR172a, bol-miR164b, bol-miR156a, bol-miR1030i) were evaluated. All miRNAs could be detected in both RC-E and DC-E. However, a significant (*p* < 0.001) decrease in concentration of miRNAs (normalized to RNA amount) was detected after GID in the bioaccessible phase. The fraction that remained stable to GID constituted less than 1% of the original concentration, with bol-miR172 showing the least number of remaining molecules (0.05%), followed by bol-miR156a (0.13%), bol-miR1030i (0.14%), and bol-miR164b (0.24%). The melt curves (Supplementary Fig. 3) had a single prominent peak for each sample at their expected value, showcasing a specific amplification.

### 3.4 Measurement of miRNA uptake by CaCo-2 cells

Experiments conducted up to this point indicated that some miRNAs from cauliflower were stable to GID. Therefore, we studied the potential uptake/association of these molecules by the gut epithelia. To this end, we focused on bol-miR172a, given that it showed the highest expression from our selected panel of miRNAs. Both polarized and non-polarized (i.e. grown in standard wells and in transwells, respectively) CaCo-2 cells were exposed to this miRNA to study its cellular uptake/association and secretion to the basolateral site.

In non-polarized cells, the detection of bol-miR172a was not possible unless the cells were exposed to said miRNA, indicating that bol-miR172a was not expressed in this cell line (Fig. 5A, Supplementary Fig. 4). Furthermore, the detection of bol-miR172a by qPCR showed a single peak in the melt curve (70 °C), indicating successful and specific amplification as already observed in previous samples (Supplementary Fig. 4). Incubation of the cells with mimic miRNA (bol-miR172a) at different concentrations (0.016 nM – 1.6 nM) indicated a dose dependent detection of said miRNAs (Fig. 5A). Alternatively, the incubation of RNA extracts (bulk total RNA) derived from raw cauliflower (300 ng RNA/μl) at different amounts (3.5–22.5 μg RNA) did not lead to successful detection (Fig. 5B). Further experiments showed that encapsulation of miRNAs using lipofectamine increased the detection of the mimic compared to non-encapsulated miRNAs (Fig. 5C). This observation was true for both mimics (39.4-fold increase in detection) and RNA extracts, with the latter being detected even with a low amount of input RNA to the supernatant (300 ng) (Fig. 5C).

**Fig. 5:**
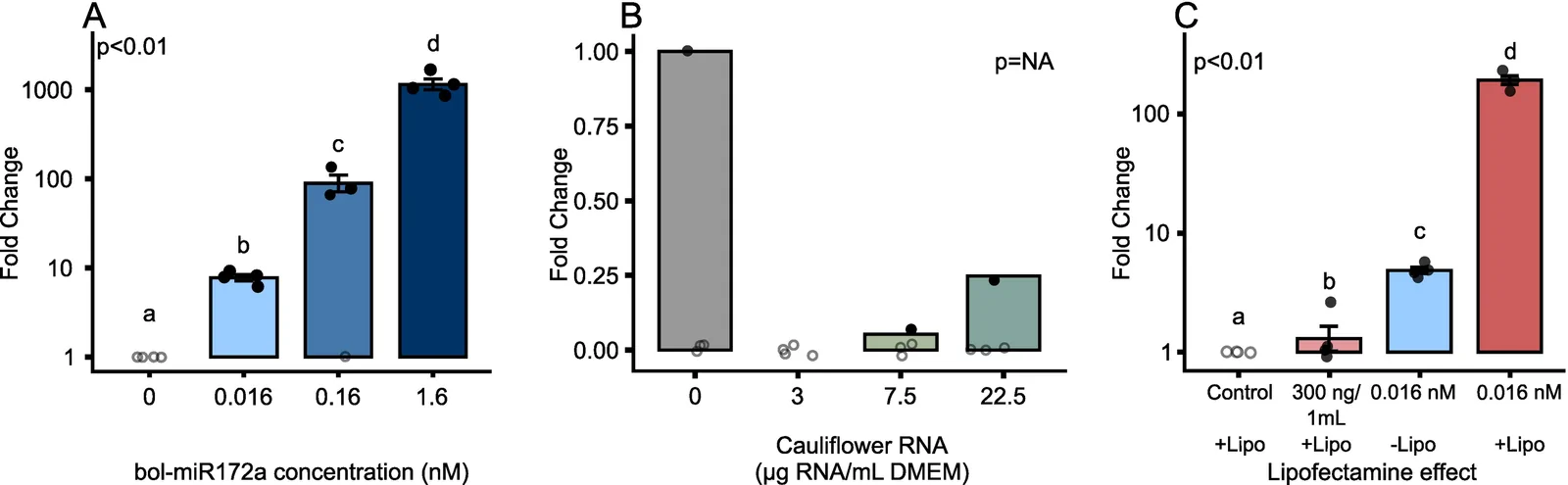
Uptake of bol-miR172a by non-polarized CaCo2 cells when incubated under different conditions. Blue represents incubation with a synthetic miRNA mimic. Grey is used for negative controls. Green is used to display incubation with raw cauliflower RNA extract (300 ng RNA/ μl). Red shows that lipofectamine has been used to encapsulate RNA. Shading indicates concentration (darker shades of a colour indicate higher concentration). Data is shown as fold change from the negative control (artificially set at Cq = 35). A: Absorption of the target miRNA (bol-miR172a) when incubated at increasing concentrations (0.016 nM–1.6 nM). B: Uptake of bol-miR172 when incubated with increasing amounts of RNA from cauliflower (3–22.5 μg). C: Detection of bol-miR172a after incubation with lipofectamine 3000. The letters displayed on top of bars indicate statistical significance between two treatments. *P*-values of the comparisons are displayed on the top left of each panel when significant. Comparisons were made using an ANOVA + Tukey's HSD. Panel B did not contain enough samples to conduct statistical testing. *N* = 4, *n* = 2. Error bars represent ± SD of the mean. (For interpretation of the references to colour in this figure legend, the reader is referred to the web version of this article.)

The cellular uptake using transwell inserts (fully polarized cells) follows the same pattern as the previous observations. On the one hand, the detection of the miRNA mimic within the RNA contents of the cell occurred under all incubation conditions and was significantly increased following longer incubation times or higher concentrations, as well as when lipofectamine was added as a nano-encapsulation agent (Fig. 6A). On the other hand, the incubation of the cell culture with extracellular vesicles (Fig. 6B) led to similar detection levels within the cells as observed for the mimic at 0.045 nM (Fig. 6). When cells were exposed to PEVs, a significant effect of concentration on miRNA detection was recorded as previously noted, however this was not observed for prolonged incubation times (Fig. 6B). When the culture was exposed to PEVs that underwent digestion, a reproducible signal within the cells was detected after more than 35 PCR cycles (>5 inverted CT). Melt curves obtained from the qPCR analysis indicated a successful and specific amplification, ruling out any artifact or primer dimers (Supplementary Fig. 5). Moreover, the standard curve of bol-miR172a primer amplification (Supplementary Table 7) remains linear until approximately 37 cycles.

**Fig. 6:**
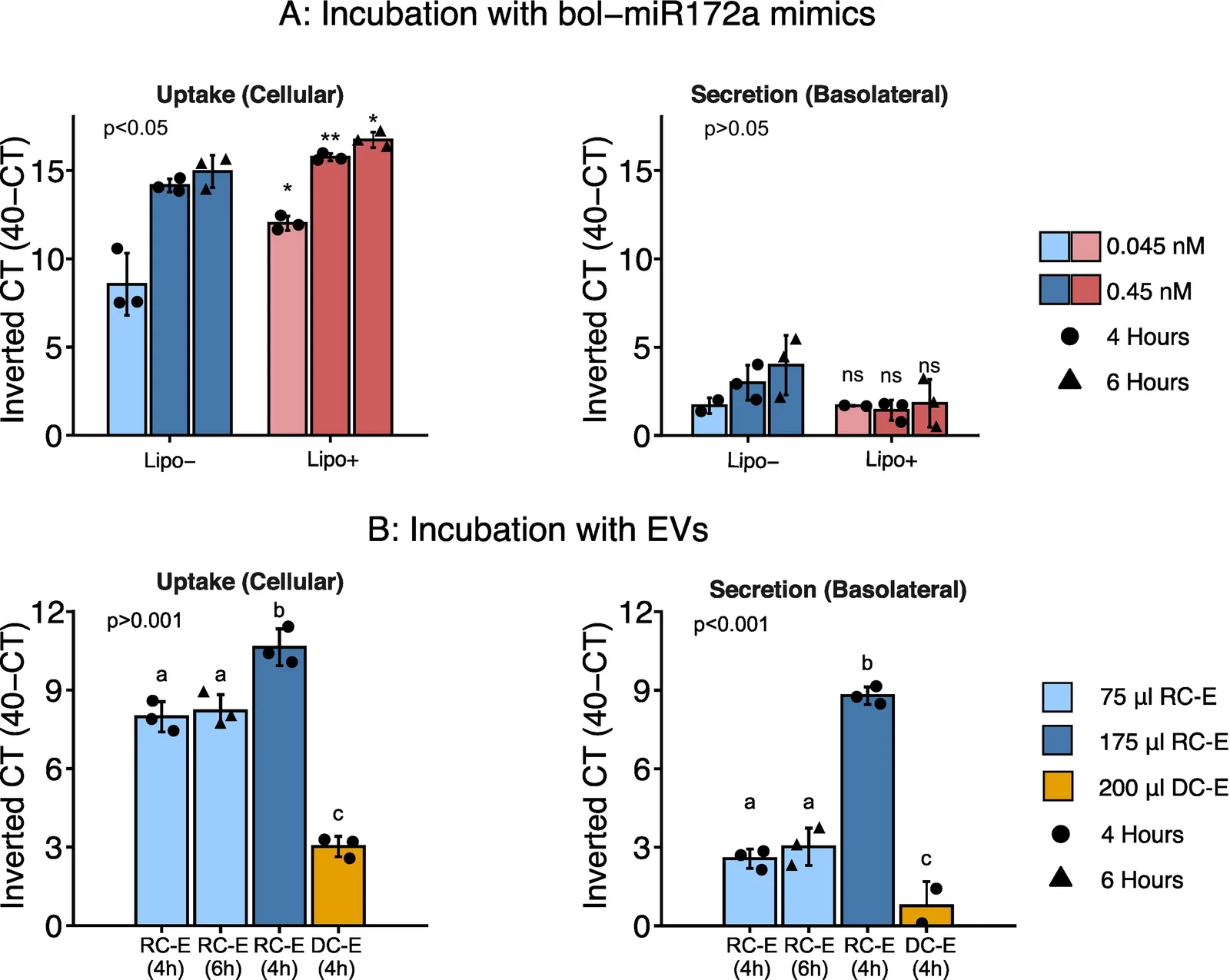
Detection of bol-miR172a in a polarized CaCo-2 cell culture, following incubation with different components. The shape of the datapoint indicates time of incubation, which was either 4 h (circle) or 6 h (triangle). Data is displayed as the inverted CT value (40-CT). Higher values represent a higher expression. A: Detection of bol-miR172a inside the cells (left) and in the basolateral site of the culture (right), following an incubation with a synthetic mimic at different concentrations (0.045 nM light shade, 0.45 nM dark shade). Blue indicates incubation without lipofectamine, red indicates use of lipofectamine during incubation. Asterisks are used to indicate significance in a comparison between incubation with and without lipofectamine in each specific condition (* *p* < 0.5, ** *p* < 0.001, ns *p* < 0.05). B: Detection of the target miRNA after incubation of the cell culture with EVs from cauliflower at different concentrations. Shading indicates concentration. Blue colors show incubation with RC-E and orange incubation with DC-E. Letters represent statistical significance across conditions in a panel. Analysis of differences was conducted by multiple Student unpaired *t*-tests followed by a Bonferroni correction in panel A and ANOVA test and a post-hoc (Tukey's) HSD in panel B. Significant differences (*p* < 0.01) were recorded for each sample between uptake and secretion, except when cells were incubated with DC-E (*p* = 0.15). *N* = 3, *n* = 2. Error bars represent ±SD. (For interpretation of the references to colour in this figure legend, the reader is referred to the web version of this article.)

### 3.5 Basolateral transport

The miRNAs secreted to the basolateral compartment of the culture were also analyzed. Our data showed that bol-miR172a was not present in the media before CaCo-2 cells were exposed to it on the apical site (Supplementary Fig. 5). The transepithelial electrical resistance (TEER) at the start of the incubation (Supplementary table 5) showed values for electrical resistance ranging from 500 to 1000 Ω*cm^−2^, as expected for a confluent monolayer (Kus et al., 2023).

The qPCR detection of bol-miR172a was delayed by multiple cycles in all samples regardless of concentration or exposure time (Fig. 4). Most importantly, bol-miR172a mimic could only be detected before 35 cycles elapsed in one condition (incubation with undigested PEVs at high concentrations). The data shows that when CaCo-2 cells were incubated with a combination of bol-miR172a and lipofectamine, the detection of the mimic associated with cells was improved (Fig. 6A). However, their secretion to the basolateral compartment remained low and was not different (*p* = 0.1) from the secretion recorded in cells exposed to the mimic without lipofectamine (Fig. 6A).

In the case of cells incubated with PEVs, we observed similar data. When CaCo-2 cells were incubated with RC-E, the detection of miRNAs associated with cellular RNA was increased at higher concentrations (Fig. 6B). Moreover, the detection of DC-E (>35 CT) associated miRNAs was significantly lower than for RC-E. Studying the basolateral compartment after incubation with PEVs indicated that RC-E incubated at high concentrations was detected via qPCR significantly earlier (Fig. 6B). Contrarily, time of exposure did not seem to affect the detection of miRNAs in the basolateral site. The values for detection of miRNAs in the basolateral site are above 35 cycles in all conditions tested except for RC-E at high concentrations. Interestingly, all conditions exhibited a large variability of detected miRNAs on the basolateral site, a variability which was not observed in the cell-associated RNA. In all cases the melt curves remained specific, with peaks at 70 °C except for the negative controls, which showed multiple peaks, indicating unspecific binding of the primer in the absence of target RNA (Supplementary Fig. 5).

## 4 Discussion

Diet-derived miRNAs have been hypothesized in the last years to be novel bioactive components of diet (Liang et al., 2012). One of their most exciting prospects is their potential to exert gene regulation in a cross-kingdom fashion in humans (Del Pozo-Acebo et al., 2021). In addition, diet-derived miRNAs have also been proposed as biomarkers of food intake, given that they meet the required criteria of stability and specificity (Cifuentes et al., 2024). Although these two important aspects have attracted the attention of researchers, large knowledge gaps persist, especially around the stability of diet-derived miRNAs to GID including the bioaccessible phase, their potential cellular uptake by the gut epithelia, and the role of PEVs in these processes (Del Pozo-Acebo et al., 2021). In this article, we highlighted that most of the total RNA contained in the food matrix is degraded during cooking, but especially during GID, which constitutes a bottleneck for RNA stability, as observed by the shift in the small RNA/total RNA ratio together with the fragment size reduction. Nevertheless, the degradation is not complete, as a fraction of miRNAs, particularly those associated with PEVs (bol-miR-172a, bol-miR-1030i and others) remain detectable in the bioaccessible phase of gastrointestinal digesta. We further showed that miRNAs are associated with CaCo-2 cells, indicative of cellular uptake or at least adsorption, possibly coupled with further transport and secretion to the basolateral site.

Recent studies have assessed some aspects of diet-derived miRNA stability either in vivo, in vitro, or in a combination of both (Diez-Sainz et al., 2024; Li, Xu, et al., 2025; Qin et al., 2022; Xing et al., 2022). Nonetheless, a complete evaluation of miRNA stability including raw vegetables moving up to final cellular uptake after GID was still missing. In this context, we have conducted experiments in which a selection of miRNAs has been evaluated at different stages of kitchen food processing such as cooking, as well as in the bioaccessible phase of GID. Cauliflower was employed as a model food matrix due to previous literature reports of diet-derived miRNAs (Liang et al., 2014).

The stability of diet-derived miRNAs was however only partial, with most of the RNA molecules being degraded (99.87%), especially (but not exclusively) during GID. In this regard, we should note that real-life cooking conditions (i.e. without nuclease free water) might increase the degrading effect observed for cooking, which was in any case quite elevated. Our experiments demonstrated that some selected miRNAs, which were associated with PEVs remained partially detectable following GID. In this regard, we believe that GID favors the stability only of small RNA molecules such as miRNAs, and out of these, only those showing the highest expression in raw vegetables, as shown by the large degrading effect (0.0033% remaining), or those that are encapsulated in extracellular vesicles (0.05–0.2% survival) (Del Pozo-Acebo et al., 2021). Interestingly, our data agrees with previously published data showing that *Brassica oleracea* miRNAs are detectable in the GID at comparable concentrations to the ones here reported (Chapado et al., 2021; López de las Hazas et al., 2024). Overall, the data indicated that longer RNA molecules were either completely degraded or broken down to a size matching that of smaller RNA molecules. Moreover, the high specificity of the primers we used allowed to rule out potential artifacts (i.e. primer dimer, contamination) for the cause of the detection of diet-derived miRNAs, as it has been argued in the past (Witwer & Zhang, 2017).

A preliminary study of the effect that different GID conditions may have over miRNA stability was also performed. Despite variability between digested replicates, we could detect miRNAs in the bioaccessible phase of GID following all tested conditions, further reinforcing the hypothesis that some miRNAs remain stable during GID. However, further insights into differences between digestion models are still needed (Cifuentes Acebal et al., 2025).

Since PEVs have been shown to be stable to GID (Wang et al., 2014) and to contain miRNAs (Lopez de Las Hazas et al., 2023), we have tried to address their role in diet-derived miRNAs stability, which remains an elusive topic (Tambaro et al., 2025). PEVs are lipophilic vesicles that should offer some protection against GID, as proposed earlier (Qin et al., 2022; Wang et al., 2014), emphasizing their potential role as delivery vehicles for a number of plant bioactives (Wang et al., 2014; Wu et al., 2024). The possibility that PEVs exert a protective effect for miRNAs has been widely discussed in literature and our data seems to support this hypothesis (Lopez de Las Hazas et al., 2023). The data highlighted in the present study shows an association between diet-derived miRNAs and PEVs, as the selected miRNAs detected after GID were enriched in the PEVs fraction of the bioaccessible GID A causal demonstration of PEVs protective role is however lacking. Furthermore, bol-miR172 stability increased by 1 order of magnitude (0.003% to 0.05%) when associated with PEVs. Although our data seems to indicate that miRNAs associated with PEVs are more stable to GID, we would like to highlight that many other aspects of miRNA chemistry and biology are known to regulate their stability during digestion. For example, plant miRNAs are known to contain a 3′-terminal 2’-O- methylation that increases their resistance to certain exonucleases (Zhang et al., 2012). Furthermore, researchers have shown in the past that both the chemical structure of plant miRNAs, as well as the food matrix can modify the resistance to GID (Qin et al., 2022; Wang et al., 2020). In addition, association of miRNAs to proteins can largely increase their half-life and resistance to degradation (Winter & Diederichs, 2011). Taken together, this data suggests that PEVs constitute one of the several drivers of diet-derived miRNAs stability to digestion.

PEVs are, however, not completely protected from digestion as shown by the decrease in total protein content (∼90% compared to RC-E), the large decrease in miRNA concentration (99.9% vs RC-E), the tendency to aggregate as shown by TEM and DLS. Also, the small droplets observed with TEM in proximity to PEVs after digestion are indicative of PEV breakdown products. Our data also suggests that DC-E is a less pure isolate than RC-E (i.e. soluble proteins may have coprecipitated with the PEVs), which may affect downstream applications. Furthermore, our analysis of PEVs has showcased that PEN1/SYP121, a typical PEVs surface marker (Pinedo et al., 2021), can be used to study PEVs, at least for cauliflower PEVs prior to digestion. However, we could not use this marker in digested PEVs, most likely due to the very low amount of protein remaining after GID, though it is also possible that the surface marker was partly denatured during GID. Potential degradation of PEV surface markers during digestion should be considered in future studies and further investigated, as it can cause disruption of the vesicle's membrane or even affect the interaction with cells (Wang et al., 2025), which might be relevant to understand their biological significance. Overall, data for PEV stability to digestion agrees with former studies, showing that these vesicles have a partial stability to GID and sometimes show a significant size increase following digestion (Jiang et al., 2024).

In this case, we believe that the apparent size increase shown by DLS is caused by the particle aggregation that TEM revealed in digested PEVs. However, our results should be interpreted with caution, as the kit we employed is known to occasionally produce lower quality EV extracts than other frequently used methods, such as ultracentrifugation or high affinity chromatography (Bruce et al., 2019; Stranska et al., 2018). Moreover, GID is known to be a complex biofluid from which isolation of molecules is not always straightforward (Cifuentes Acebal et al., 2025). Thus, a question remains on the isolation efficiency of digested PEVs and their cargo, as highlighted by the decrease of the PEV solution purity. Regardless of said limitations, which might affect the final number of PEVs isolated, we have demonstrated that at least some PEVs and their cargo are stable to GID. Furthermore, it remains to be understood which plant fraction of miRNAs is encapsulated in PEVs. A hallmark paper on plant miRNAs in PEVs established a decade ago that miRNAs are selectively packaged into PEVs. However, this does not mean a complete depletion of PEV-enriched miRNAs in other cellular locations of the plant (Baldrich et al., 2019).

Having studied the stability of miRNAs and PEVs following GID, we considered the uptake of diet-derived miRNAs by the gut epithelia. The data showed that bol-miR172a, a plant miRNA, did not occur naturally in CaCo-2. Furthermore, our results indicated that the uptake of miRNAs by CaCo-2 cells, a common model of the small intestinal epithelium (Vahid et al., 2024), is a process likely mediated by concentration and time. However, with the current methodological set-up it was not possible to rule out completely that the detected miRNA was bound to the cellular membrane instead of being taken up by the cells, though the washing steps should have reduced this risk (Rogers et al., 2014). Both encapsulated miRNAs (i.e. extracellular vesicles and lipofectamine), as well as free miRNAs, appeared to be highly associated with cellular RNA, which is in accordance with cellular uptake up. However, future studies should perform additional mechanistic demonstrations of cellular uptake. Furthermore, encapsulation, either artificially via liposomes (lipofectamine), or naturally via PEVs, seemed to improve the detection of miRNAs associated with cells (40-fold increase) compared to free miRNAs. It is likely that these vesicles were taken up via endocytosis compared to free miRNAs, as reported earlier (Li, Yi, et al., 2025). How these values obtained in vitro relate to physiological concentrations is a matter of discussion. The concentrations of mimic miRNAs and bulk RNA were selected to match those in 50 mg of raw cauliflower. Although the concentrations reaching the gut epithelia would be much smaller than those in the raw vegetables as we have demonstrated due to the degrading effect of GID, we believe the absolute number of miRNAs might be somewhat in the same range. Especially if we consider that a typical dietary portion of vegetables may contain more than a hundred grams of food (Eldridge et al., 2025).

The data regarding the basolateral transport of miRNAs is more complex, in part due to a combination of large intrasample variability and low concentrations being secreted. In most instances, the target miRNA could only be detected above 35 PCR cycles, a traditional threshold for quantification (de Ronde et al., 2017). Given that most results were above this threshold, caution is advised in the interpretation of the data. However, the reproducibility of the data and specificity of the primer as indicated by the melt curves suggest limited and inconsistent secretion, which will need further future validation. Thus, we identified a possible role of PEVs in miRNA secretion into the basolateral lumen, which also warrants further confirmatory experiments. Interestingly, the negative charge of PEVs might have played a role in the increased secretory profile as it had previously been suggested that nanoparticles associate with different cellular transport pathways depending on their surface charge (Bannunah et al., 2014). Moreover, we observed a statistical trend, showing that lipofectamine decreased the secretion of miRNAs by CaCo-2 cells, likely facilitating uptake, but then fostering intracellular retention, an observation that would match Lipofectamine's positive charge expected behavior (Bannunah et al., 2014). However, this deserves further extensive validation before reaching definitive conclusions.

All in all, some technical limitations persisted in the current study, and further work remains to be carried out with respect to revealing the role that different digestive conditions (pH, enzyme concentration) may have on miRNA stability, or the study of PEVs uptake and basolateral transport in a reproducible and systematic manner. For example, an improved characterization of PEVs following digestion is much desired. In this regard, our results on particle concentration performed by MADLS provide an estimate rather than an exact value (Vogel et al., 2021), and would greatly benefit from other orthogonal methods frequently employed, such as nano particle tracking analysis (NTA) (Welsh et al., 2024). Moreover, DLS is not the most robust tool to work with when a polydisperse sample is present as is the case for DC-E, as it might overestimate the particle size (Welsh et al., 2024). In the same regard, TEM is known to cause changes in particle size during the preparatory dehydration of the sample for visualization, leading to size underestimations (Welsh et al., 2024). Thus, other techniques (e.g. Cryo-EM) might be more appropriate to quantify the size and lipid bilayer of PEVs, especially after digestion (Welsh et al., 2024).

Other conflicting aspects in this regard include the lack of detection of *SYSP121* or *TET8* in the digested PEVs. We believe that the less pure PEVs isolate, together with a lower protein concentration post-digestion might have been underlying causes. However, degradation of the surface/transmembrane proteins during GID should not be ruled out. Therefore, future experiments should consider the inclusion of cytosolic PEV proteins (Welsh et al., 2024), which might be more stable to GID. Moreover, thorough work to demonstrate a causal link between the presence of PEVs and diet-derived miRNA stability is lacking. It could be considered to isolate PEVs and treat them with RNAses to show their protective effect. Also, the combination of RNAses with detergents that disrupt the EVs would further elucidate their role in miRNAs stability to digestion.

Regarding the cellular work, further in-depth analysis using confocal microscopy (e.g. PKH26 dying of PEVs) and in-situ hybridization could demonstrate the internalization of miRNAs and PEVs into the cells. In addition, although nano-encapsulation using lipofectamine might provide interesting data at the current stage of research, it should be considered that this is not a physiological condition and data on this topic should be considered with caution. For example, it is well understood that simple changes in the vesicle presented to endothelial cells, such as the surface charge, can significantly affect their uptake and secretion (Bannunah et al., 2014). Furthermore, higher concentrations of PEVs should be incubated with CaCo-2 cells to ensure that stronger qPCR signals can be detected, to fully elucidate the miRNA secretion. Finally, the current study relied heavily on qPCR for multiple experiments. However, GID samples might occasionally be difficult to work with in molecular biology environments as shown by mildly and strongly digested samples. Thus, caution is advised on the interpretation of this data.

Furthermore, a critical aspect for the xenomiR hypothesis is to verify whether diet-derived miRNAs can have a bioactive effect on humans, given the rather small concentrations that they reach the gut epithelia and further downstream (Li, Xu, et al., 2025). In this regard, miRNAs are known to be stable in circulation over longer periods than other RNA biotypes (Aryani & Denecke, 2015; Li et al., 2021) due to their binding to carrier proteins (Katayama et al., 2025), EV encapsulation (Koberle et al., 2013) and intrinsic features related to their size (i.e. lower stochastic binding of RNAses) (Katayama et al., 2025). Therefore, we hypothesize that any effect of diet-derived miRNAs may be linked to a long-term dietary intake, rather than a onetime exposure, which would probably fail to reach the needed concentrations to exert any genetic regulation. However, in vitro experimentation has shown that diet-derived miRNAs are capable of modulating human gene expression (López de las Hazas et al., 2024; Zhang et al., 2012), which together with the present data opens the door to the development of future nutraceuticals with health promoting properties.

In conclusion, this manuscript showcases, by means of cauliflower, that miRNAs contained in food matrices can survive cooking and be detected in the bioaccessible phase of GID to a certain extent. Nonetheless, a large fraction of miRNAs (99.9%) is lost, especially during GID. We have shown that the miRNAs detected after digestion can be found in the PEV fraction, reinforcing the hypothesis that suggests a protective role of these vesicles (Lopez de Las Hazas et al., 2023). We have also shown that miRNAs, especially when encapsulated in PEVs or other lipophilic vesicles such as lipofectamine, can become associated, i.e. absorbed or at least adsorbed with the gut epithelia and maybe transported to the basolateral site. Future studies should focus on exploring the mechanisms governing the uptake and secretion mechanisms of PEVs in the gut epithelia.

## CRediT authorship contribution statement

**Miguel Cifuentes-Acebal:** Writing – original draft, Methodology, Formal analysis, Data curation, Conceptualization. **Adria Vilalta:** Writing – review & editing, Methodology, Formal analysis. **Ximena Terra:** Writing – review & editing, Investigation. **Yvan Devaux:** Writing – review & editing, Supervision, Resources, Methodology. **Torsten Bohn:** Writing – review & editing, Supervision, Resources, Project administration, Methodology, Conceptualization.

## Declaration of generative AI and AI-assisted technologies in the writing process

During the preparation of this work the author(s) used Gemini 3 and Claude Sonnet in order to improve the code used for statistical analysis and plotting (adding comments, reducing redundancies, debugging, etc.). After using this tool/service, the author(s) reviewed and edited the content as needed and take(s) full responsibility for the content of the published article.

## Funding source

This work was supported by the Luxembourgish Research Fund (FNR) (PRIDE21/16749720/NEXTIMMUNE2). Adrià Vilalta received a doctoral research grant from the FI-SDUR programme (2022 FISDU 00245) of the Generalitat de Catalunya.

## Declaration of competing interest

The authors declare the following financial interests/personal relationships which may be considered as potential competing interests: Miguel Cifuentes-Acebal reports financial support was provided by Luxembourg National Research Fund. Adria Vilalta reports financial support was provided by Government of Catalonia. If there are other authors, they declare that they have no known competing financial interests or personal relationships that could have appeared to influence the work reported in this paper.

## Data Availability

The authors have set up a GitHub repository that contains all the data used in the article as well as the scripts used to generate the figures and the statistical testing. To access the repository please use the following link: https://github.com/miguel1cifu/miRNA_survival_supplementary

## Appendix A Supplementary data

Supplementary material 1 List of Supplementary Figures

Supplementary material 2 List of Supplementary Tables
