# Supplementary material for: A fraction of diet-derived microRNAs present in plant extracellular vesicles remain detectable after gastrointestinal digestion and shows epithelial uptake in a CaCo-2 cell model
Source: Food Chem (Oxf). 2026 Sep 18;13:100450. doi: 10.1016/j.fochms.2026.100450 (PMC13641049; doi:10.1016/j.fochms.2026.100450)
Supplement: Supplementary file 1 [file mmc1.docx]

Supplementary Figure 1. A-D: Absorption spectra (blue line) from RNA extracts (N=4) derived from Raw Cauliflower (RC), Cooked Cauliflower (CC), Digested Cauliflower (DC), and Cooked and Digested Cauliflower (DCC) respectively. Red dotted lines indicate absorbance at 230 and 260 nm respectively. E: Detection of bol-miR164b RT-qPCR in the previously described samples (n=2). F: Detection of bol-miR1030i through RT-qPCR n=2. N=4 Error bars represent ± SD of means. Blank columns and/or missing data points represent no detection. Data in panels E and F is shown as the raw CT value for detection. The values displayed result from averaging the technical replicates. The tables underneath the barplots detail the characteristics of each condition.

Supplementary Figure 2. Absorption of RNA extracted from cauliflower digested under weak and strong digestive conditions. The red dotted lines indicate the absorption at 230 and 260 nm respectively. N=4

Supplementary Figure 3. Additional data regarding extracellular vesicle characterization. A: Total protein content of the EVs plotted together with the BCA standard curve. B: Quantification of EVs total protein content in a PVDF membrane stained with no-stain labelling reagent, on the left (blue) of the gel undigested EVs, on the right (orange) of the gel digested EVs. Top and bottom figures show the same gel after a shorter (top) and a longer (bottom) exposure time. C: Same membrane as previous panel, incubated with anti-TET8 (29KDa, not visible) and anti-SYP121 (37KDa, lowest black band). D-G: Melt curve data for the qPCR reactions shown in Figure 2. Blue indicates a reaction for R-EV samples and D-EV are shown in orange.

Supplementary Figure 4, Melt curve data obtained through qPCR for bol-mir-172a following its uptake by CaCO-2 as shown in Figure 3. The figure also contains the melt curves for negative controls

Supplementary Figure 5. Melt curve data obtained through qPCR for bol-mir-172a following its uptake and basolateral secretion by CaCO-2 as shown in Figure 4. The figure also includes melt curves for negative controls

Supplementary Figure 6. Detection of two spike-in controls in qPCR experiments. The threshold values for exclusion of a sample are defined in supplementary table 7. In short, values above 20.5 CT for cel-miR-39-3p (control for RNA extraction) or 24.0 for UniSp6 (RT spike in control). The dots in black are those samples above their respective thresholds. Samples not passing the CT were repeated from the failed stage. If the experiment failed twice the sample was excluded from analysis.

Supplementary Figure 7. Detection of two spike-in controls in qPCR experiments corresponding to figure 6. The threshold values for exclusion of a sample are defined in supplementary table 7. In short, values above 20.5 CT for cel-miR-39-3p (control for RNA extraction) or 24.0 for UniSp6 (RT spike in control). Data for UniSp6 in the cellular cDNA contents is not available. All data points for cel-miR-39-3p are above the threshold of 20.5 when RNA was extracted from cell culture media.
